# Supplementary figures and images for: Comparing Intraperitoneal and Intravenous Personalized ErbB2CAR-T for the Treatment of Epithelial Ovarian Cancer
Source: Biomedicines. 2022 Sep 7;10(9):2216. doi: 10.3390/biomedicines10092216 (PMC9496506; doi:10.3390/biomedicines10092216)

Supp Figure S1

supplementary

A.

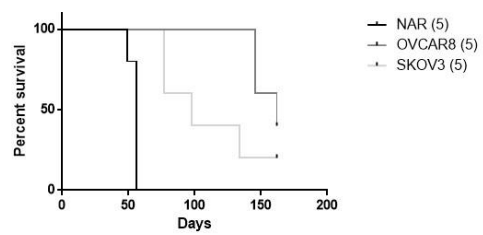

Supplement: Supplementary file 1 [file biomedicines-10-02216-s001.zip › biomedicines-1861896-supplementary.pdf]
